# Rubrene-Directed Structural Transformation of Fullerene (C_60_) Microsheets to Nanorod Arrays with Enhanced Photoelectrochemical Properties

**DOI:** 10.3390/nano12060954

**Published:** 2022-03-14

**Authors:** Ning Chen, Pengwei Yu, Kun Guo, Xing Lu

**Affiliations:** State Key Laboratory of Materials Processing and Die & Mould Technology, School of Materials Science and Engineering, Huazhong University of Science and Technology, 1037 Luoyu Road, Wuhan 430074, China; nchen@hust.edu.cn (N.C.); yupw@hust.edu.cn (P.Y.)

**Keywords:** fullerene, rubrene, nanorod arrays crystal transformation, charge transfer

## Abstract

One-dimensional (1D) nanostructures possess huge potential in electronics and optoelectronics, but the axial alignment of such 1D structures is still a challenging task. Herein, we report a simple method that enables two-dimensional (2D) C_60_ microsheets to evolve into highly ordered nanorod arrays using rubrene as a structure-directing agent. The structural transformation is accomplished by adding droplets of rubrene-*m*-xylene solution onto C_60_ microsheets and allowing the *m*-xylene solvent to evaporate naturally. In sharp contrast, when rubrene is absent from *m*-xylene, randomly oriented C_60_ nanorods are produced. Spectroscopic and microscopic characterizations collectively indicate a rather plausible transformation mechanism that the close lattice match allows the epitaxial growth of rubrene on C_60_ microsheets, followed by the reassembly of dissolved C_60_ along the aligned rubrene due to the intermolecular charge-transfer (CT) interactions, leading to the formation of ordered nanorod arrays. Due to the aligned structures and the CT interactions between rubrene and C_60_, the photocurrent density of the nanorod arrays is improved by 31.2% in the UV region relative to the randomly oriented counterpart. This work presents a facile and effective strategy for the construction of ordered fullerene nanorod arrays, providing new ideas for the alignment of fullerene and other relevant organic microstructures.

## 1. Introduction

The peculiar and fascinating properties of one-dimensional (1D) nanostructures have enabled the widespread applications in diverse fields such as electronics, photonics, energy, and so forth [1]. Of particular interest are 1D nanostructures made of π-conjugated molecules due to their mechanical flexibility, solution processability, and minimized defects [2,3]. In light of this, 1D organic nanostructures hold great promise in next-generation electronic and optoelectronic devices. For the purpose of on-demand photons/electrons transport on the macroscale, it is strongly desired that the 1D nanostructures are unidirectionally oriented. Otherwise, the anisotropic feature disappears when they are randomly aligned [4,5,6,7,8].

Several approaches have been developed for aligning 1D organic nanostructures into highly ordered arrays, including Langmuir–Blodgett (LB), dip-coating, spin-coating, templating, and printing techniques [9,10,11,12,13,14,15]. However, these methods often involve tedious and complicated procedures to fabricate organic molecules. For example, in spin coating or printing methods, regioselective substrate patterning by altering the surface wettability is required to deposit the precursor solution onto these modified regions. After solvent evaporation, organic building blocks assemble into 1D aligned nanostructures [12,15]. The LB method involves the introduction of external forces for aligning 1D nanostructures, whereas in the templating method, the utilized templates need to be removed in the production of 1D nanostructural arrays [13,14]. Therefore, facile and efficient synthesis of ordered alignment of 1D nanostructures remains a challenging task.

As a carbon allotrope, fullerene C_60_ is a well-defined π-conjugated molecule that consists of all sp^2^-hybridized carbon atoms. The unique intermolecular π–π interaction, moderate highest occupied molecular orbital–lowest unoccupied molecular orbital (HOMO–LUMO) energy gap, and special electronic structure indicate C_60_ as a promising building block of high-performance organic semiconductor devices, such as field effect transistors and photodetectors [16,17,18]. To date, several methodologies, such as liquid-liquid interfacial precipitation (LLIP), solvent evaporation, and solvent vapor annealing, have been developed for the synthesis of 1D, two-dimensional (2D), and three-dimensional (3D) fullerene micro-/nanostructures [19,20,21,22,23]. Similar to other organic molecules, efforts have also been devoted to fabricating aligned fullerene nanostructures using the LB or template methods [14,24,25,26,27]. Recently, the epitaxial growth of the aligned nanorod superstructures was realized through a simple structural transformation from 2D crystals [28,29,30]. For example, Hu et al. reported the crystal transformation from 2D benzoperylene microsheets to benzoperylene-1,3-dicyanotetrafluorobenzene nanorod meshes due to the close lattice match between the two crystals [29]. Moreover, Chen et al. reported the crystal transformation of C_60_ from hexagonal microplates to nanorod meshes [31]. The lattice matching between the 2D microsheets and the as-transformed nanorod meshes lays the foundation of such epitaxial transformation. However, although the obtained superstructures present a cross-linked nano-mesh morphology, the axial alignment of nanorods is still hard to achieve due to the lack of suitable 2D microsheets. Fortunately, various 2D fullerene microstructures can be easily obtained, providing more chances to realize the preparation of fullerene nanorod arrays with axial alignment [23,32,33].

**Figure 1:**
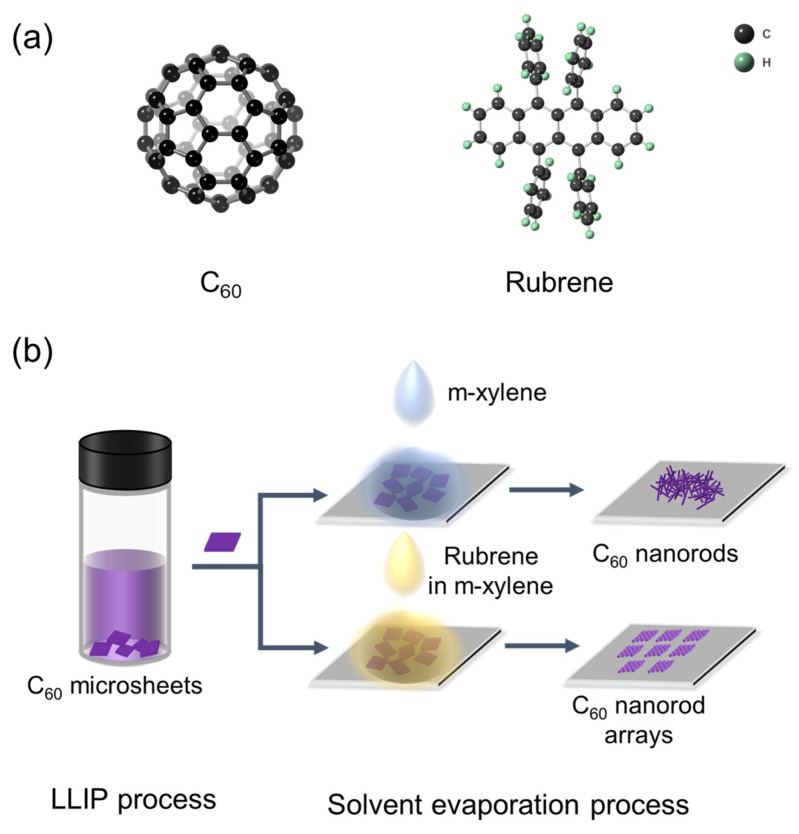
(**a**) Molecular structures of C_60_ and rubrene. (**b**) Schematic illustration of the fabrication processes of C_60_ nanorods and C_60_ nanorod arrays.

Inspired by the above results, we then assume that using an electron-donor material as the structure-directing agent may lead to the aligned orientation upon the transformation of 2D fullerene crystals, due to the intermolecular interactions and lattice matching. We herein present an effortless approach to promote the transformation from 2D fullerene microsheets to nanorod arrays with the assistance of rubrene as a structure-directing agent (Figure 1). Specifically, C_60_ rhombic microsheets were first prepared, onto which a solution of rubrene in *m*-xylene was dropped. Upon *m*-xylene evaporation, re-dissolved C_60_ microsheets evolve into highly ordered nanorod arrays. In contrast, randomly oriented C_60_ nanorods were formed in the absence of rubrene. Detailed characterizations reveal that the epitaxial growth of rubrene on C_60_ microsheets leads to the orientation of rubrene. The intermolecular charge transfer interaction further drives the reassembly of C_60_ molecules around the rubrene to eventually form the nanorod arrays. Such arrays show superior photoelectrochemical properties to the randomly aligned counterpart, due to their highly ordered orientation, high crystallinity, and improved charge carrier transport upon the usage of rubrene.

## 2. Materials and Methods

Materials: Pristine C_60_ was synthesized by the direct-current arc discharge method and isolated using high-performance liquid chromatography. Figure S1 displays the laser desorption ionization time-of-flight (LDI-TOF) mass spectrum of as-obtained C_60_ (>99.9%). Rubrene powder (>98%) was purchased from Beijing InnoChem Science & Technology Co., Ltd., Beijing, China. Analytical-grade toluene, *m*-xylene, isopropyl alcohol (IPA), and tert-butanol (TBA) were purchased from Beijing Chemical Ltd., China. All the chemicals were used without further purification.

Preparation of C_60_ microsheets (**C_60_MSs**): **C_60_MSs** were synthesized using the liquid-liquid precipitation (LLIP) method [34]. Typically, 12.5 mg of C_60_ was dissolved in 10 mL of toluene by ultrasonication for 2 h to obtain a C_60_-toluene solution (1.25 mg mL^−1^). One milliliter of the C_60_-toluene solution was rapidly injected into a 10 mL glass vial preloaded with 3 mL of TBA, resulting in a turbid mixture. **C_60_MSs** were formed after keeping the mixture for 12 h without any disturbance at 25 °C, and then recovered by centrifugation and vacuum drying at 60 °C for 12 h.

Preparation of C_60_-rubrene nanorod arrays (**C_60_-RNRAs**) and C_60_ nanorods (**C_60_NRs**): **C_60_-RNRAs** and **C_60_NRs** were prepared by the structural transformation of **C_60_MSs**. Typically, 0.5 mg of **C_60_MSs** was dispersed in 20 μL of IPA and transferred onto a silica substrate (3 cm × 3 cm) to form a uniform **C_60_MSs** thin film upon IPA evaporation. The thin film was dried under vacuum at 60 °C for 8 h to remove residual IPA. Subsequently, 60 μL of rubrene-*m*-xylene solution (1 mg mL^−1^) was dribbled onto the silica substrate to cover the **C_60_MSs** film and then naturally dried at 25 °C to generate **C_60_-RNRAs**. In contrast, **C_60_NRs** were prepared by adding 60 μL of *m*-xylene without rubrene. All the experimental steps were conducted at 25 °C in air atmosphere.

Photoelectrochemical measurements: Photoelectrochemical measurements of the samples were conducted through a CHI 660E electrochemical workstation (CH Instruments Inc., Shanghai, China) at 25 °C. A 500 W xenon lamp (Saifan Photoelectronic Co., Beijing, China) was utilized as the light source with filters of 400–760 nm and 350 nm to obtain the light in the corresponding ranges. A standard three-electrode system was utilized for the measurements. Two milligrams of **C_60_MSs**, **C_60_NRs**, or **C_60_-RNRAs** was dispersed in 4 mL of deionized water and gently agitated to form uniform suspensions, respectively. Next, 2 μL of the as-obtained suspensions were dribbled on an indium tin oxide (ITO) glass (5 mm × 60 mm) to form thin films (5 mm × 5 mm), which were used as the working electrodes. A Pt wire and an Ag/AgCl electrode were used as the counter and reference electrodes, respectively. All the measurements were performed in 0.1 M KCl at a bias voltage of 1 V. The electrolyte solution was bubbled with Ar for 30 min to remove the oxygen, and all the tests were conducted in an Ar atmosphere.

Characterizations: The morphology of the above samples was observed using a Nova NanoSEM 450 field-emission scanning electron microscope (FESEM, FEI, Eindhoven, Netherlands). Transmission electron microscopic (TEM) images were obtained by a Talos F200X G2 electron microscope (FEI, Hillsboro, OR, USA). Powder X-ray diffraction (PXRD) patterns were collected on an Empyrean diffractometer (PANalytical B.V., Eindhoven, Netherlands) with Cu Kα radiation. The d spacings of the planes were calculated according to XRD patterns and the Bragg equation (2dsinθ = nλ). Raman and photoluminescence spectra were measured on a Horiba JobinYvon LabRAM HR800 spectrometer (Horiba Group, Paris, France) using a 532 nm green laser. Fourier-transform infrared spectroscopy (FT-IR) was performed on a Bruker VERTEX 70 FTIR spectrometer (Bruker, Karlsruhe, Germany). The mass spectrum of C60-RNRAs was acquired on a Bruker MICROFLEX MALDI-TOF. Ultraviolet-visible (UV-vis) spectra (Bruker, Karlsruhe, Germany) were recorded on a SolidSpec-3700 spectrometer (Shimadzu, Kyoto, Japan). Thermogravimetric (TG) analysis was carried out in N2 at a heating rate of 10 °C/min on a TGA8000 (PerkinElmer, WA, USA).

## 3. Results and Discussion

**Figure 2:**
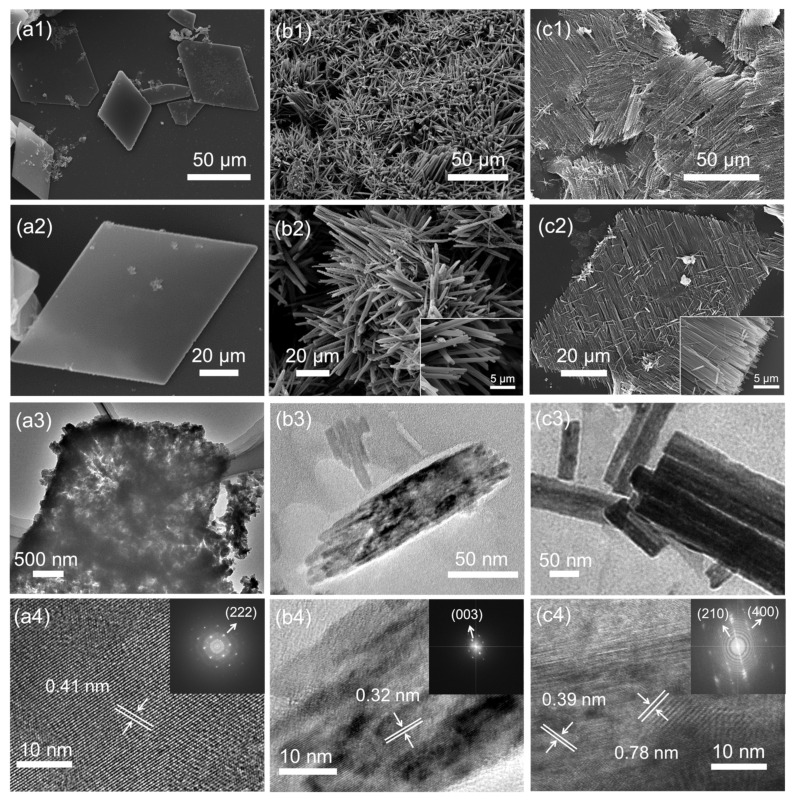
SEM, TEM, and HRTEM images of **C_60_MSs** (**a1**–**a4**), **C_60_NRs** (**b1**–**b4**), and **C_60_-RNRAs** (**c1**–**c4**). Insets of (**b2**,**c2**) are magnified SEM images. Insets of (**a4**,**b4**,**c4**) are the corresponding electron diffraction patterns (The arrows point to the corresponding planes).

**C_60_MSs** prepared by the LLIP method present a rhombic shape (Figure 2(a1,a2)). TEM image reveals the loosely packed character of **C_60_MSs** (Figure 2(a3)). HRTEM image of **C_60_MSs** shows its crystalline structure with a lattice spacing of 0.41 nm, which matches well with that of the (222) plane of the face-centered cubic (fcc) C_60_ (*a* = 1.410 nm) [35,36], indicating that the ordered stacking of C_60_ molecules along the [111] direction forms the rhombus sheets, whose top/bottom surfaces are exposed by the (222) plane (Figure 2(a4)). As a good solvent of C_60_, *m*-xylene is known to generate rod-shaped structures by the LLIP [37,38]. In light of this, *m*-xylene is adopted to trigger the potential microsheets-to-nanorods transformation. Figure 2(b1,b2) show that **C_60_MSs** are indeed successfully transformed into **C_60_NRs**, but the alignment of nanorods is completely random. Moreover, the aggregated nanorods in **C_60_NRs** are observed under TEM (Figure 2(b3)), which displays a lattice spacing of 0.32 nm, corresponding to the (003) plane of the hexagonal close packed (hcp) C_60_ (*a* = 2.376, *c* = 1.008 nm) (Figure 2(b4)) [37].

We then select rubrene as a structure-directing agent, and the reasons are discussed in the following context. After adding a solution of rubrene in *m*-xylene instead of only *m*-xylene to **C_60_MSs**, **C_60_MSs** are readily evolved into **C_60_-RNRAs**, as discerned in Figure 2(c1,c2) and Figure S2, showing unidirectionally oriented nanorod arrays. More specifically, the orientation of nanorods is parallel to one of the diagonals that connects two obtuse angles of the rhomboid. After sonicating the resulting **C_60_-RNRAs** in water for 30 min, no other types of structures but dispersed nanorods are observed (Figure S3), indicating the complete transformation of all the microsheets into nanorods rather than only the surface part. This is supported by the TEM image shown in Figure 2(c3). HRTEM image of one single nanorod in **C_60_-RNRAs** presents lattice spacings of 0.78 and 0.39 nm, which match well with those of the (210) plane of the hcp C_60_ and the (400) plane of the orthorhombic rubrene (*a* = 1.540 nm, *b* = 0.748 nm, *c* = 2.792 nm), respectively (Figure 2(c4)) [39]. These results reveal that crystalline rubrenes are embedded into the nanorods and stacked separately with the C_60_ molecules inside **C_60_-RNRAs**.

**Figure 3:**
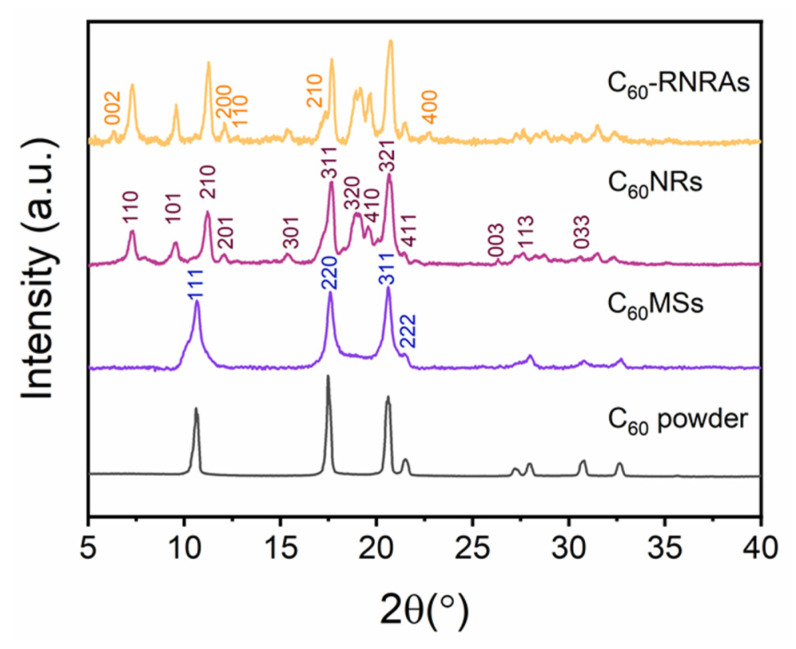
Powder XRD patterns of C_60_ powder, **C_60_MSs**, **C_60_NRs,** and **C_60_-RNRAs**.

Powder XRD patterns of relevant samples are shown in Figure 3. **C_60_MSs** present the same fcc structure as the pristine C_60_ powder [35], whereas **C_60_NRs** formed by the transformation of **C_60_MSs** after the evaporation of *m*-xylene exhibit a different hcp structure (*a* = 2.376 nm, *c* = 1.008 nm). Moreover, **C_60_-RNRAs** include both the hcp **C_60_NRs** and the orthorhombic structured rubrene (*a* = 1.560 nm, *b* = 0.736 nm, *c* = 2.768 nm), again indicating the existence of rubrene crystals inside **C_60_-RNRAs**. Note that the hcp structure of C_60_ and orthorhombic structure of rubrene are also obtained by simply evaporating the C_60_-*m*-xylene and rubrene-*m*-xylene solutions, respectively (Figure S4) [37,40]. Moreover, according to the full-width at half-maximum (FWHM) values for (210) and (311) peaks of hcp C_60_ in **C_60_NRs** and **C_60_-RNRAs** (Figure S5), smaller FWHM values are obtained for **C_60_-RNRAs**, suggesting their higher crystallinity than **C_60_NRs** [41].

**Figure 4:**
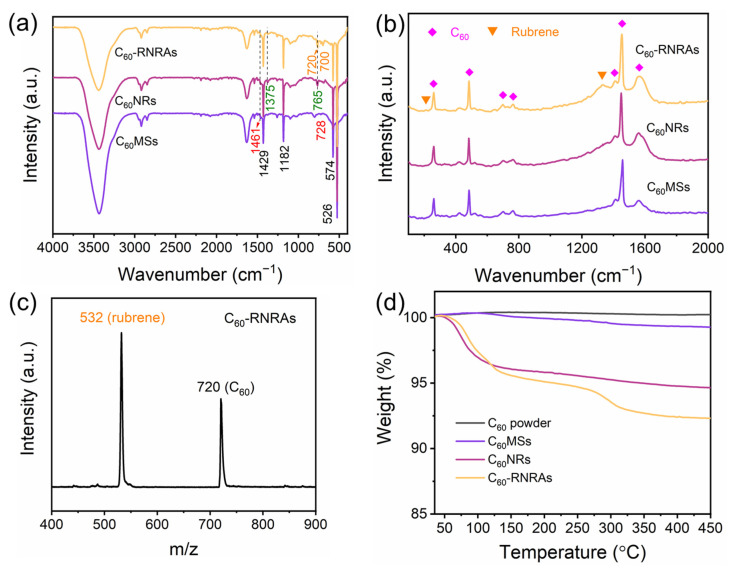
(**a**) FT-IR and (**b**) Raman spectra of **C_60_MSs**, **C_60_NRs**, and **C_60_-RNRAs**, (**c**) LDI-TOF mass spectrum of **C_60_-RNRAs**, and (**d**) TG analysis of C_60_ powder, **C_60_MSs**, **C_60_NRs**, and **C_60_-RNRAs**.

FTIR spectra of **C_60_MSs**, **C_60_NRs,** and **C_60_-RNRAs** are shown in Figure 4a. All three samples show typical absorption bands of C_60_ at 1429, 1182, 574, and 526 cm^−1^ [42]. The absorption bands of **C_60_MSs** at 1461 and 728 cm^−1^ can be assigned to the C-H bending vibration in the benzene ring and C-H wagging vibration in methyl of toluene, respectively, indicating the presence of toluene molecules inside **C_60_MSs** [43]. The spectra of **C_60_NRs** and **C_60_-RNRAs** also contain the major absorption band of toluene at 1461 cm^−1^, indicating toluene molecules remain in the lattices even after the structural transformation. Moreover, the absorption bands at 1375 and 765 cm^−1^ of **C_60_NRs** and **C_60_-RNRAs** can be ascribed to C-C stretching vibration and C-H bending vibration in the benzene ring of *m*-xylene, respectively, suggesting the existence of *m*-xylene molecules in **C_60_NRs** and **C_60_-RNRAs** [44]. More importantly, additional FT-IR bands at 720 and 700 cm^−1^ in **C_60_-RNRAs** are observed. According to the FT-IR spectrum of pristine rubrene (Figure S6a), these two bands shall be assigned to specific vibrations of rubrene molecules, which implies the presence of rubrene in **C_60_-RNRAs** [45]. Raman spectra are also acquired to provide further information. As shown in Figure 4b, additional Raman peaks at 211 and 1328 cm^−1^ detected in **C_60_-RNRAs**, in comparison to **C_60_NRs** and **C_60_MSs**, can be assigned to rubrene (Figure S6b) [46]. LDI-TOF mass spectrum of **C_60_-RNRAs** dissolved in CS_2_ also clearly shows peaks at *m/z* of 532 and 720, originating from rubrene and C_60_, respectively (Figure 4c). These results unambiguously confirm the presence of rubrene in **C_60_-RNRAs**.

TG analysis of C_60_ powder, **C_60_MSs**, **C_60_NRs**, and **C_60_-RNRAs** are then carried out to determine the solvent contents of these materials (Figure 4d). Compared to C_60_ powder, the weight loss of **C_60_MSs** beginning at approximately 115 °C is about 0.8 wt%, which corresponds to the contained toluene molecules. In contrast, weight losses of **C_60_NRs** and **C_60_-RNRAs** begin at approximately 55 °C, and amount to 4.8 wt% and 5.2 wt%, respectively, equal to the total amount of entrapped toluene and *m*-xylene molecules. Therefore, the content of *m*-xylene is much higher than that of toluene. In addition, **C_60_-RNRAs** show a further weight loss of 3.4 wt% above the temperature of 250 °C, which is confidently ascribed to the removal of rubrene molecules (Figure S6c).

**Figure 5:**
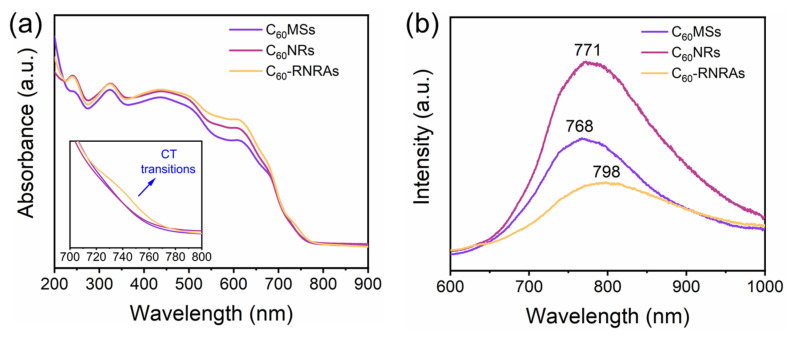
(**a**) UV-vis absorption spectra and (**b**) PL spectra of **C_60_MSs**, **C_60_NRs**, and **C_60_-RNRAs**.

The UV-vis absorption and photoluminescence (PL) spectra of these samples in the steady-state mode were then performed to investigate the intermolecular interactions between C_60_ and rubrene molecules. Figure 5a displays the corresponding absorption spectra of these samples. In contrast to **C_60_MSs** and **C_60_NRs**, a new absorption peak appears at approximately 730 nm for **C_60_-RNRAs**. Given that C_60_ and rubrene are well known as electron-accepting and electron-donating materials, respectively, this new peak could be attributed to the charge transfer (CT) transitions between C_60_ and rubrene as also reported by previous studies [16,47,48]. Notably, the CT interaction between C_60_ and rubrene is energetically favorable (Figure S7) [16,49]. Figure 5b shows the PL spectra of the three samples under study. Clearly, **C_60_NRs** exhibit the enhanced PL intensity compared to **C_60_MSs**, indicating that the transformation from microsheets to nanorods enhances the PL intensities due to the morphological evolution and the increase of entrapped solvents [22,50]. Moreover, **C_60_-RNRAs** show an obvious decrease in PL intensity along with a red shift of the PL peak (798 nm) relative to those of **C_60_MSs** (768 nm) and **C_60_NRs** (771 nm), which are frequently observed in organic cocrystals due to the CT interactions [16,47]. These results again corroborate the occurrence of CT interactions between C_60_ and rubrene within **C_60_-RNRAs** [51,52,53,54].

**Figure 6:**
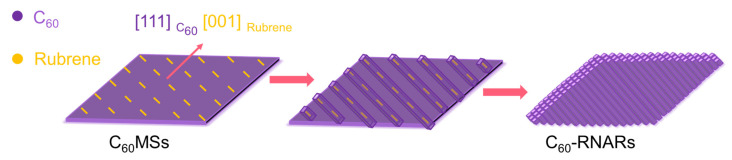
Schematic illustration of the transformation from **C_60_MSs** to **C_60_-RNARs**.

Based on the above results, we further investigated the transformation mechanism from **C_60_MSs** to **C_60_NRs** and **C_60_-RNRAs**. According to the XRD pattern of rubrene crystals prepared by evaporating the rubrene-*m*-xylene solution, rubrene of the same orthorhombic structure to the pristine rubrene powder is obtained. However, the most intense diffraction peak changes from the (400) peak for crystallized rubrene to the (002) peak for original powder, indicating the growth of rubrene along the [100] direction when *m*-xylene evaporates (Figure S4). HRTEM image of the rubrene film prepared by drop-drying the rubrene-*m*-xylene solution on the silica substrate displays a clear lattice spacing of 0.39 nm, which derives from the (400) plane of the orthorhombic rubrene, confirming again that the rubrene molecules crystallize along the [100] orientation with the gradually evaporation of *m*-xylene (Figure S8). The XRD and HRTEM results of rubrene crystals obtained by evaporating the rubrene-*m*-xylene solution are in line with the rubrene in **C_60_-RNRAs**, indicating the same growth behavior of rubrene in **C_60_-RNRAs** with the above rubrene crystals. Notably, the (222) plane of fcc C_60_ that grows along the [111] orientation has an interplanar spacing (d_222_ = 0.41 nm) close to that of the (400) plane of orthorhombic rubrene (d_400_ = 0.39 nm), thus affording the desired lattice matching for epitaxial growth of rubrene [29,30,31]. Therefore, as depicted in Figure 6, when the rubrene-*m*-xylene solution drops on **C_60_MSs**, the epitaxial growth of [001] oriented rubrene molecules on the [111] orientation of **C_60_MSs** is first achieved as *m*-xylene evaporates. Afterwards, the emerging intermolecular CT interactions between C_60_ and rubrene mediate the oriented growth of re-dissolved C_60_ molecules as *m*-xylene gradually evaporates, eventually leading to the formation of crystalline rubrene inside C_60_ nanorod arrays aligned parallelly to the obtuse diagonal of **C_60_MSs**.

Effects of the rubrene concentration in *m*-xylene on the formation of nanorod arrays were also studied. When the rubrene concentration decreased from 1.0 to 0.5 mg mL^−1^, the same preparation steps would give rise to nanorod arrays with a narrowed width and without the rhombic morphology compared to **C_60_-RNRAs**, indicating that 0.5 mg mL^−1^ rubrene concentration is too low to induce adequate interactions between rubrene and C_60_ (Figure S9a). However, the increase of rubrene concentration to 2.0 mg mL^−1^ results in the formation of nanorod arrays with reduced ordering, which is probably due to the local aggregation of excessive amounts of rubrene (Figure S9b). Accordingly, the rubrene concentration is a key parameter that regulates the formation of aligned nanorod arrays.

To further confirm the versatility of this rubrene-directed **C_60_MSs** to **C_60_-RNARs** conversion strategy, we then conduct control experiments by simply changing the shape or crystal phase of C_60_ microsheets. The rectangle C_60_ microsheets with a fcc structure (*a* = 1.410 nm) were also synthesized using benzene/TBA as the good/poor solvents (the concentration of C_60_-benzene solution is 2 mg mL^−1^, a volume ratio of 1:3, 25 °C), respectively (Figure S10). The rectangle microsheets were then treated using *m*-xylene and rubrene-*m*-xylene solutions (1 mg mL^−1^). As a consequence, randomly oriented nanorods were formed only when treated by *m*-xylene, whereas the rectangle nanorod arrays were still formed in the presence of rubrene. Furthermore, hcp C_60_ microsheets (*a* = 1.025 nm, *c* = 1.072 nm, denoted as hcp 1, Figure S12) were prepared from the CCl_4_/IPA system (the concentration of C_60_-CCl_4_ solution is 0.4 mg mL^−1^, a volume ration of 1:3, 5 °C) [23]. However, these hcp microsheets were transformed into randomly oriented nanorods when treated with a rubrene-*m*-xylene solution, due undoubtedly to the lattice mismatch between hcp C_60_ microsheets and orthorhombic rubrene. Even so, the orthorhombic rubrene still exists in the hcp structured C_60_ microsheets (denoted as hcp 2, Figure S12).

**Figure 7:**
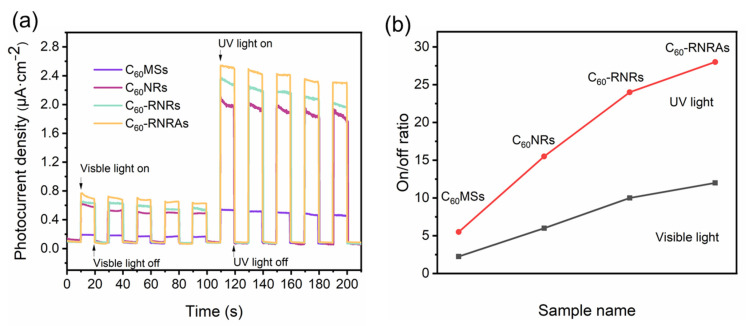
(**a**) Typical photocurrent response curves and (**b**) on-off ratios of **C_60_MSs**, **C_60_NRs**, and **C_60_-RNRAs** under visible light (400–760 nm) or UV light (350 nm) irradiation. Conditions: 0.1 M KCl aqueous solution at 1 V bias voltage.

Both C_60_ and rubrene are organic molecules with π-conjugated systems, showing great potential in the field of optoelectronics [55,56,57]. Therefore, we measured the photocurrent response curves of thin films on ITO glasses composed of **C_60_MSs**, **C_60_NRs**, and **C_60_-RNRAs** to explore their photoelectrochemical properties. To evaluate the effect of rubrene on the photoelectrochemical properties of C_60_ nanorods, **C_60_-RNRAs** were dispersed in deionized water and then ultrasonicated for 30 min to obtain the scattered C_60_-rubrene nanorods (denoted as **C_60_-RNRs**). All the samples display fast and uniform photocurrent responses under both visible and UV light irradiation during every on-off cycle (Figure 7) and are more sensitive to UV light than visible light due to their good absorption properties in the UV region (Figure 5a).

Of all the samples examined here, **C_60_MSs** show the lowest photocurrent density, which is due to their smaller specific surface area relative to the nanorods, thereby decreasing the utilization efficiency of light. In addition, the π-π stacking of C_60_ along the 1D nanorods facilitated the charge transportation [58,59,60]. Thus, photoelectrochemical properties of the nanorods are improved compared to microsheets. Moreover, **C_60_-RNRs** present a higher photocurrent density than **C_60_NRs**. A plausible explanation is a faster recombination of photocarriers due to the intermolecular CT interactions between C_60_ and rubrene in **C_60_-RNRs** [61,62,63]. More interestingly, the photocurrent density and the on-off ratio of **C_60_-RNRAs** are further improved compared to **C_60_-RNRs** (improved by 31.2% and 13.5% in the UV region and 21% and 7.1% in the visible region, compared with **C_60_NRs** and **C_60_-RNRs**, respectively), indicating that the larger surface area of highly ordered nanorod and excellent crystallinity of **C_60_-RNRAs** afford more efficient light utilization relative to that of randomly oriented nanorods [58,64]. Additionally, similar to gratings or photonic crystals, ordered nanostructures exhibit stronger absorption and less scattering properties relative to randomly aligned counterparts, therefore the photocurrent density of highly ordered **C_60_-RNRAs** is enhanced [64]. These results suggest the potential applications of well-aligned nanorod arrays in the fields of photodetectors and organic solar cells.

## 4. Conclusions

We have developed a rubrene-directed strategy with high simplicity and efficiency to enable the structural transformation from 2D C_60_ microsheets to highly ordered nanorod arrays. The key to the transformation is rooted in the lattice matching between the fcc structured C_60_ and orthorhombic structured rubrene. Rubrene crystals are first epitaxially grown on the C_60_ microsheets and then mediate the subsequent unidirectional growth of re-dissolved C_60_ molecules along a specific orientation, due to the intermolecular charge transfer interactions between C_60_ and rubrene molecules. Due to the highly oriented structures and favorable intermolecular interactions from the donator/acceptor pair, these ordered nanorod arrays show remarkable enhancement of the photoelectrochemical properties compared to randomly oriented C_60_ nanorods, indicating their potential application in the field of photodetectors. This work provides an in-depth understanding of the rational design and fabrication of fullerene and other ordered organic superstructures.

## Data Availability

The data presented in this study are available on reasonable request from the corresponding author.

## Supplementary Materials

The following supporting information can be downloaded at: https://www.mdpi.com/article/10.3390/nano12060954/s1, Figure S1: LDI-TOF mass spectrum of pristine C_60_ powder. Figure S2: SEM image of C_60_-RNRAs at different magnifications. Figure S3: SEM and TEM images of the detached C_60_ nanorods from C_60_ nanorod arrays that are subject to water and ultrasonication for 20 min. Figure S4: PXRD patterns of rubrene powder and rubrene crystals. Figure S5: FT-IR spectrum, Raman spectrum, and TG curve of rubrene. Figure S6: Full width at half maximum values for 210 and 311 peaks of C_60_NRs and C_60_-RNRAs obtained from their XRD patterns. Figure S7: Band diagrams of rubrene and C_60_. Figure S8: TEM and HRTEM images of the rubrene film. Figure S9: SEM images of as-obtained C_60_ nanorod arrays using the concentration of rubrene in *m*-xylene of 0.5 and 2.0 mg mL^−1^. Figure S10: SEM images of (a) rectangle C_60_ microsheets, (b) C_60_ nanorods, and (c) rectangle C_60_-rubrene nanorod arrays. Figure S11: SEM images of (a) hexagonal C_60_ microsheets, (b) C_60_ nanorods, and (c) C_60_-rubrene nanorod arrays. Figure S12: XRD patterns of rectangle C_60_ microsheets, rectangle C_60_-rubrene nanorod arrays, hexagonal C_60_ microsheets, and C_60_-rubrene nanorods.
