# Supplementary material for: Rubrene-Directed Structural Transformation of Fullerene (C60) Microsheets to Nanorod Arrays with Enhanced Photoelectrochemical Properties
Source: Nanomaterials (Basel). 2022 Mar 14;12(6):954. doi: 10.3390/nano12060954 (PMC8953273; doi:10.3390/nano12060954)
Supplement: Supplementary file 1 [file nanomaterials-12-00954-s001.zip › nanomaterials-1570834-supplementary.pdf]

nanomaterials

MDPI

Supplementary Materials of

Rubrene-Directed Structural Transformation of Fullerene (C_{60}) Microsheets to Nanorod Arrays with Enhanced Photoelectrochemical Properties

Ning Chen, Pengwei Yu, Kun Guo* and Xing Lu*

State Key Laboratory of Materials Processing and Die & Mould Technology, School of Materials Science and Engineering, Huazhong University of Science and Technology, 1037 Luoyu Road, Wuhan, 430074 China.
* Correspondence: authors: K Guo (Email: guok@hust.edu.cn) and X Lu (Email: lux@hust.edu.cn)

Citation: Chen, N.; Yu, P.; Guo, K.; Lu, X. Rubrene-Directed Structural Transformation of Fullerene (C_{60}) Microsheets to Nanorod Arrays with Enhanced Photoelectrochemical Properties. *Nanomaterials* **2022**, *12*, 954. https://doi.org/10.3390/nano12060954

Academic Editor: Jun-ho Yum

Received: 10 January 2022
Accepted: 4 February 2022
Published: 14 March 2022

Publisher's Note: MDPI stays neutral with regard to jurisdictional claims in published maps and institutional affiliations.

Copyright: © 2022 by the authors. Submitted for possible open access publication under the terms and conditions of the Creative Commons Attribution (CC BY) license (http://creativecommons.org/licenses/by/4.0/).

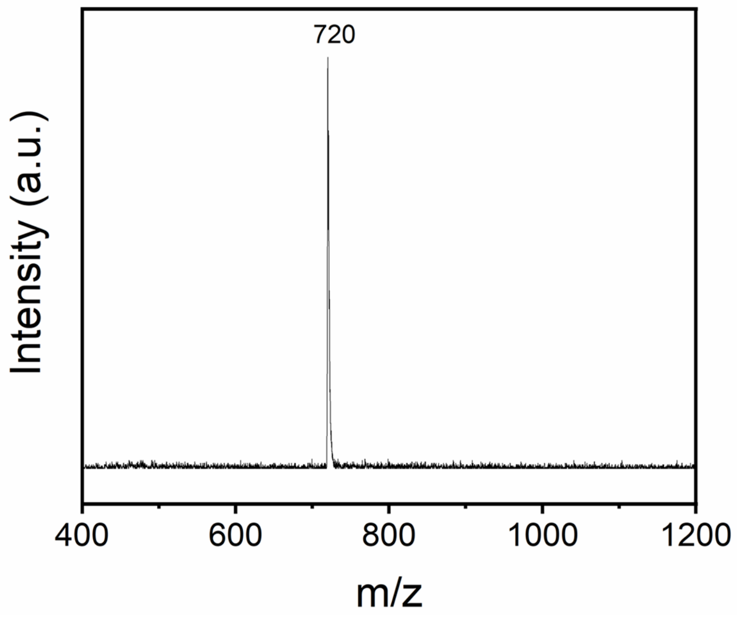

Figure S1. LDI-TOF mass spectrum of pristine C_{60}.

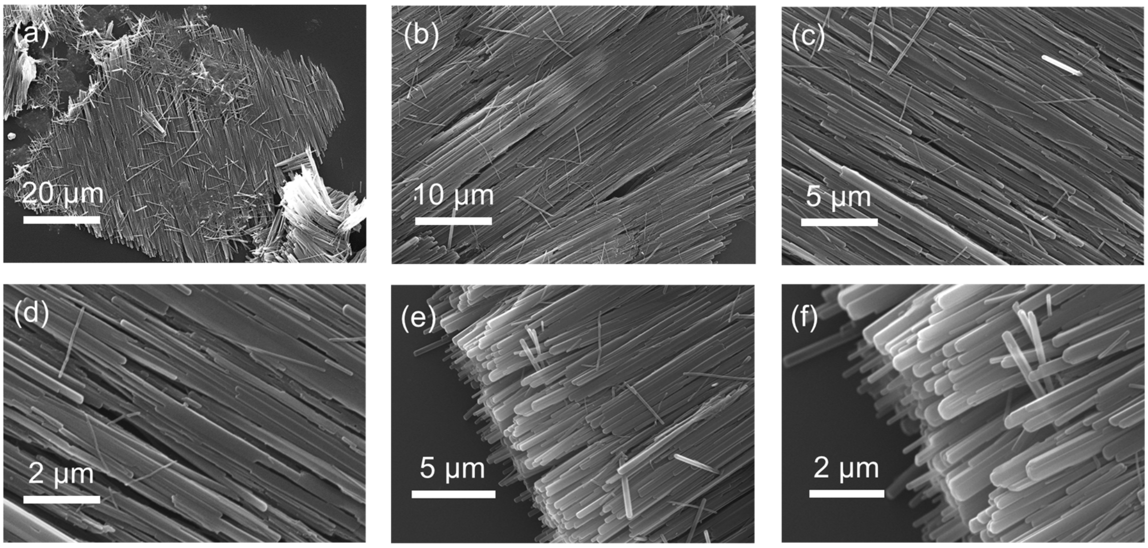

Figure S2. SEM images of $\mathbf{C_{60}}$**-RNRAs** at (**a**–**c**) low and (**d**–**f**) high magnifications.

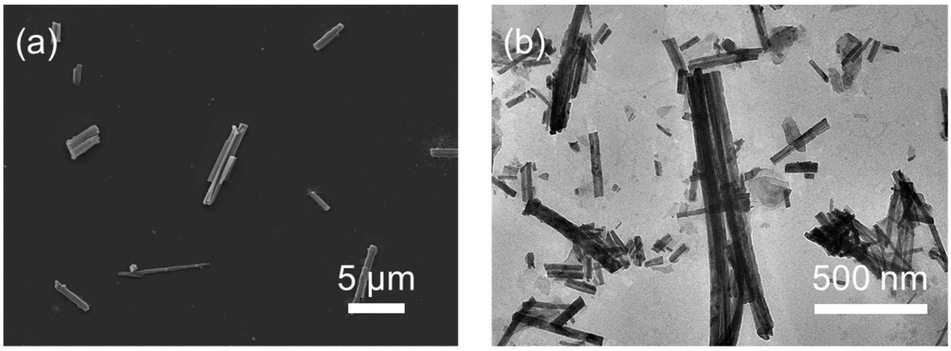

Figure S3. (**a**) SEM and (**b**) TEM images of the detached C_{60} nanorods from $\mathbf{C_{60}}$**-RNRAs** that are subject to ultrasonication in water for 20 min.

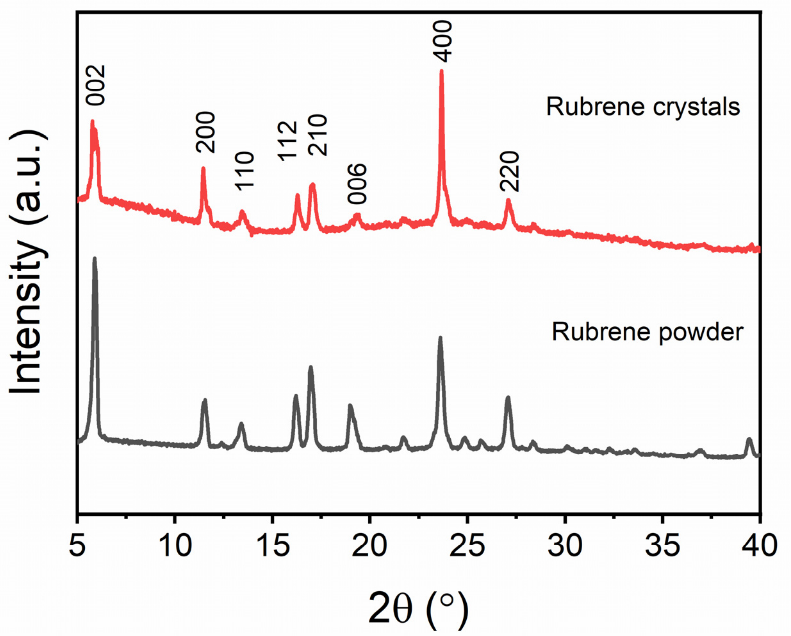

Figure S4. Powder XRD patterns of rubrene powder and rubrene crystals prepared by evaporating a rubrene-*m*-xylene solution.

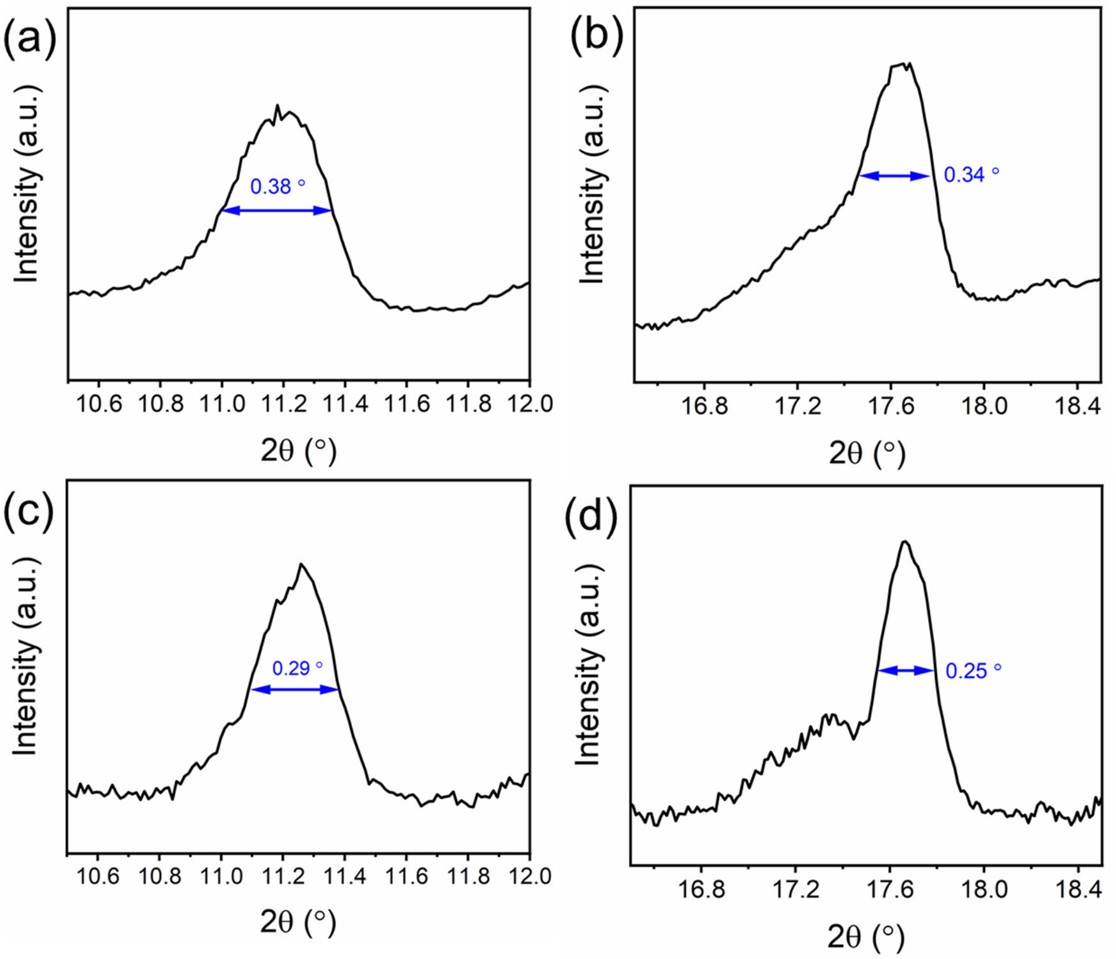

Figure S5. Full width at half maximum (FWHM) values for (**a**) 210 and (**b**) 311 peaks of **C_{60}NRs** and (**c**) 210 and (**d**) 311 peaks of **C_{60}-RNRAs** obtained from their XRD patterns.

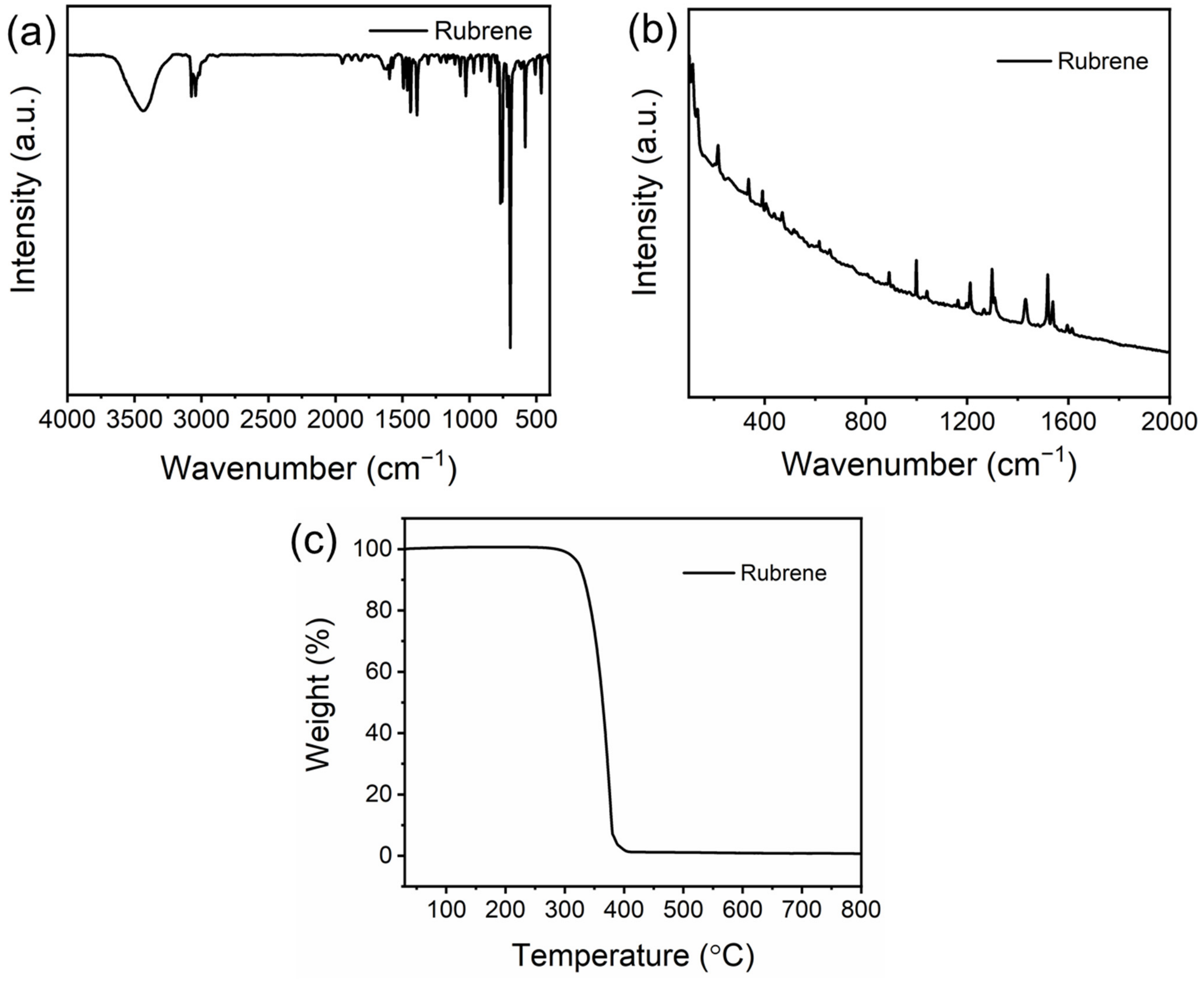

Figure S6. (**a**) FT-IR spectrum, (**b**) Raman spectrum, and (**c**) TG curve of rubrene.

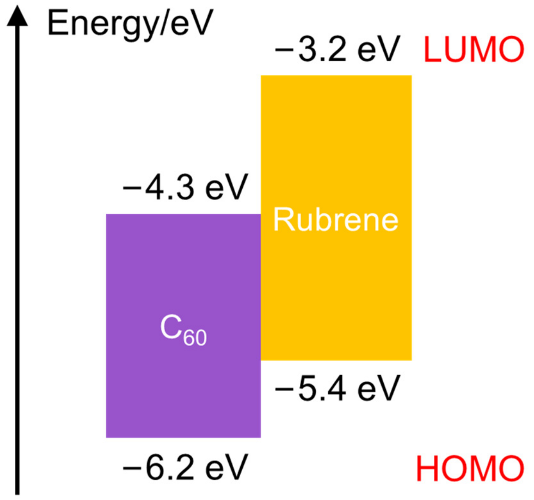

Figure S7. Highest occupied molecular orbital–lowest unoccupied molecular orbital (HOMO–LUMO) energy gaps of rubrene and C_{60}.

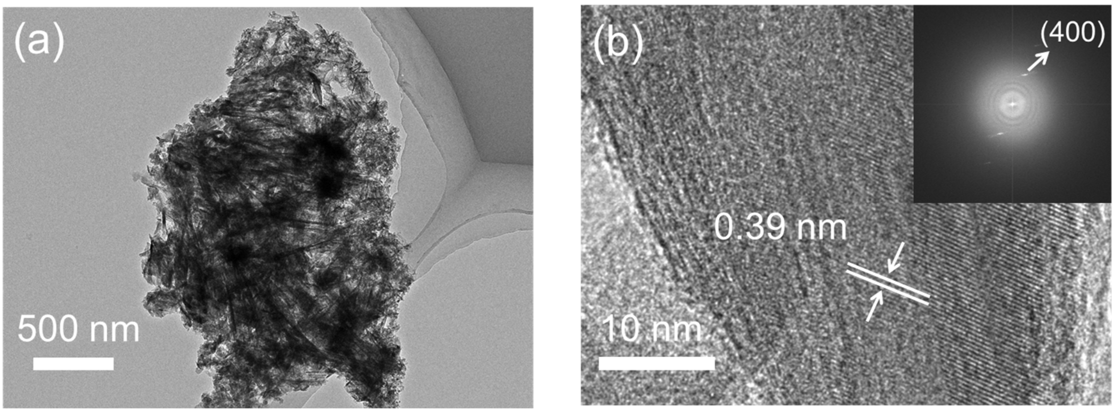

Figure S8. (**a**) TEM and (**b**) HRTEM images of the rubrene film. Inset of (**b**) is the corresponding diffraction pattern (The arrow in the inset image denotes the corresponding plane).

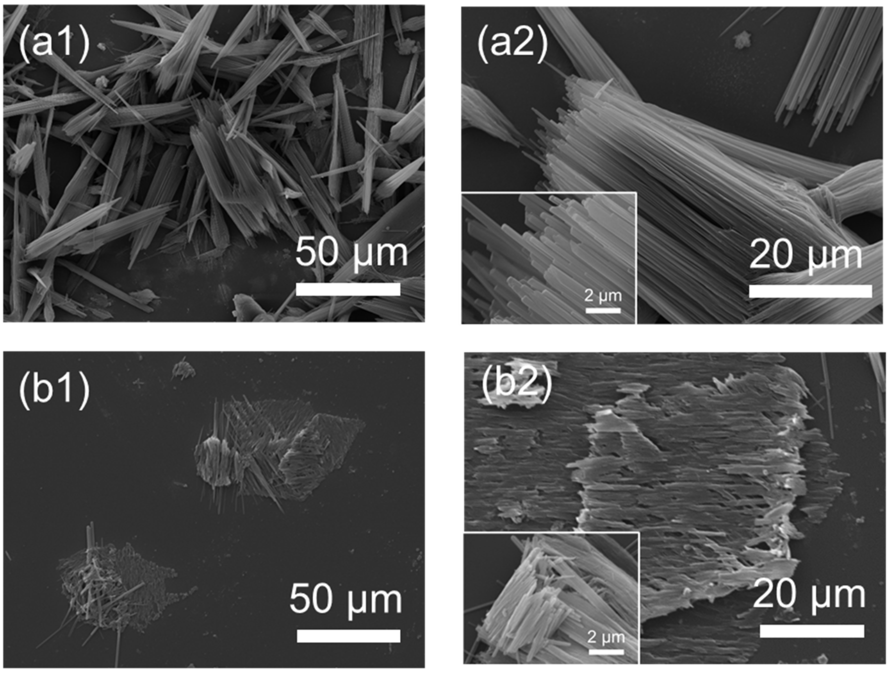

Figure S9. SEM images of as-obtained C_{60} nanorod arrays using (**a**) 0.5 and (**b**) 2.0 mg mL^{-1} rubrene in m-xylene solutions. Insets are corresponding magnified images.

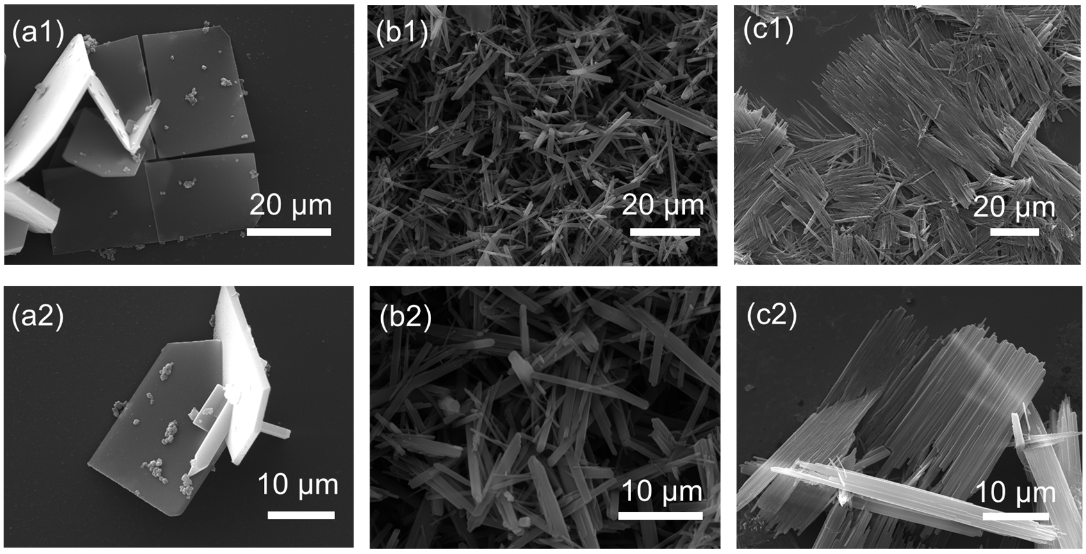

Figure S10. SEM images of (**a**) rectangle C60 microsheets, (**b**) C60 nanorods, and (**c**) rectangle C60-rubrene nanorod arrays.

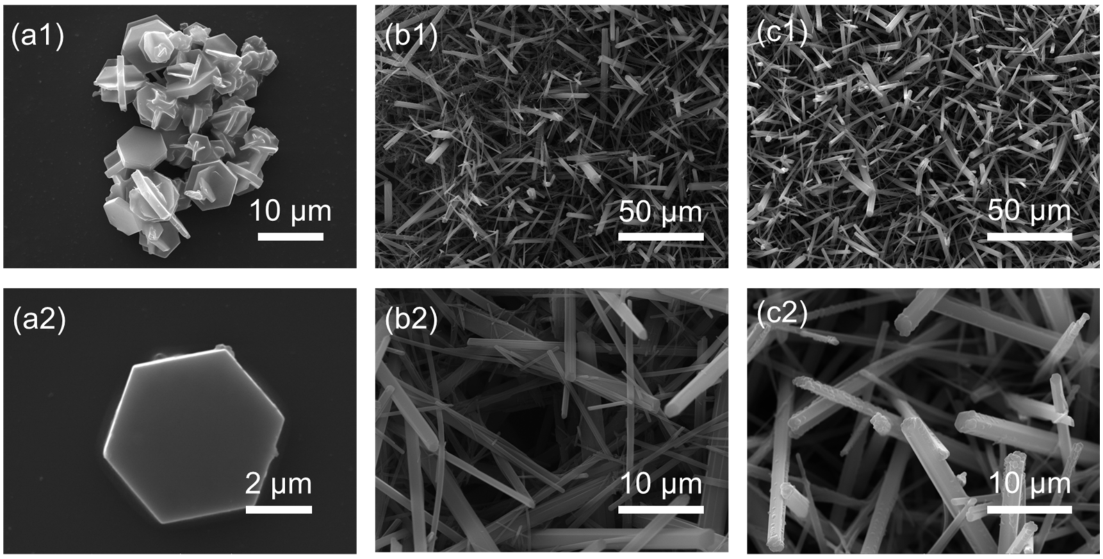

Figure S11. SEM images of (**a**) hexagonal C_{60} microsheets, (**b**) C_{60} nanorods, and (**c**) C_{60}-rubrene nanorod arrays.

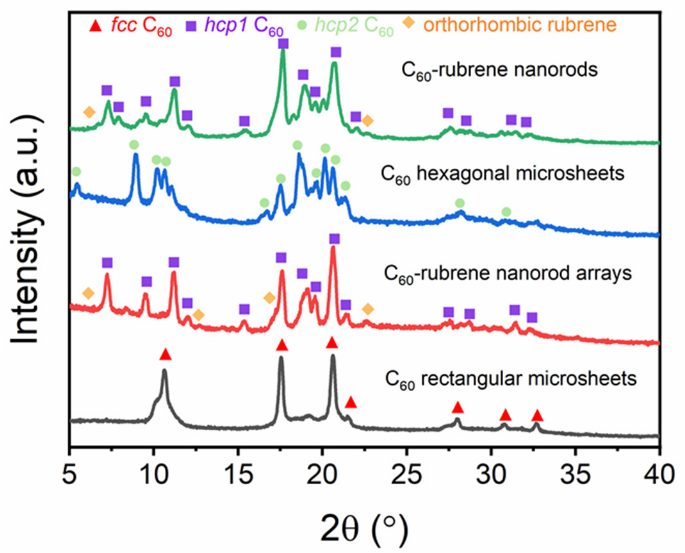

Figure S12. XRD patterns of rectangle C_{60} microsheets, rectangle C_{60}-rubrene nanorod arrays, hexagonal C_{60} microsheets, and C_{60}-rubrene nanorods.
